# The costs and benefits of positive illusions

**DOI:** 10.3389/fpsyg.2015.00859

**Published:** 2015-06-30

**Authors:** Spyros Makridakis, Andreas Moleskis

**Affiliations:** ^1^Economics and Business, Neapolis University of Pafos, PafosCyprus; ^2^Decisions Sciences, INSEAD, FontainebleauFrance; ^3^Decision Sciences, INSEAD, SingaporeSingapore

**Keywords:** illusion of control, illusions, better that average symptom, cost/benefits of illusions, gambling fallacy, self rated health (SRH), planning fallacy, preventive medicine

## Abstract

Positive illusions are associated with unrealistic optimism about the future and an inflated assessment of one’s abilities. They are prevalent in normal life and are considered essential for maintaining a healthy mental state, although, there are disagreements to the extent to which people demonstrate these positive illusions and whether they are beneficial or not. But whatever the situation, it is hard to dismiss their existence and their positive and/or negative influence on human behavior and decision making in general. Prominent among illusions is that of control, that is “the tendency for people to overestimate their ability to control events.” This paper describes positive illusions, their potential benefits but also quantifies their costs in five specific fields (gambling, stock and other markets, new firms and startups, preventive medicine and wars). It is organized into three parts. First the psychological reasons giving rise to positive illusions are described and their likely harm and benefits stated. Second, their negative consequences are presented and their costs are quantified in five areas seriously affected with emphasis to those related to the illusion of control that seems to dominate those of unrealistic optimism. The costs involved are huge and serious efforts must be undertaken to understand their enormity and steps taken to avoid them in the future. Finally, there is a concluding section where the challenges related to positive illusions are noted and directions for future research are presented.

Positive illusions are associated with unrealistic optimism about the future and an inflated assessment of one’s abilities (Kruger et al., 2009). Such illusions are prevalent in normal life and can be considered useful in some cases for maintaining a healthy mental state (Taylor and Brown, 1988, 1994), although, there is disagreement to the extent to which people demonstrate these positive illusions and whether they are beneficial or not (Colvin and Block, 1994). But whatever the situation, it is hard to dismiss their existence and their positive and/or negative influence on human behavior and decision making in general. Prominent among illusions is that of control that is defined by Langer (1975) as *“an expectancy of a personal success probability higher than the objective probability would warrant”* (p. 313). She also claims that *“while people may pay lip service to the concept of chance they behave as though chance events are subject to control"* (p. 311).

This article describes positive illusions, talks about their potential benefits but also quantifies their costs in five specific fields (gambling, stock and other markets, new firms and startups, preventive medicine and wars). It is organized into three parts. First the psychological reasons giving rise to positive illusions are described and their likely harm and benefits stated. Second, theirnegative consequences are presented and their costs are quantified in five areas that are seriously affected. Finally, there is a concluding section where the challenges related to positive illusions are noted and directions for future research are presented.

The psychological literature is full of examples showing that human judgment is generally optimistic due to overconfidence while considering insufficiently, or even ignoring negative consequences (McKenna, 1993; Kruger, 1999). The end result is that people tend to underestimate the chance of failure whereas overestimating the likelihood of success. This becomes more evident when evaluating future plans or actions requiring forecasts about forthcoming, uncertain events whose benefits are overestimated while their risks are undervalued (e.g., the planning fallacy below, Lovallo and Kahneman, 2003). A critical question is if it is possible to achieve some psychologically optimal level by seeing things as only slightly better than they really are in order to benefit from illusions while avoiding the serious risks of acting on false, unrealistic assumptions. To answer this question we need to explore both the advantages and damages associated with positive illusions and take into account the costs involved.

## Positive Illusions

People view themselves more positively compared to how they judge others. Furthermore, people also consider themselves less negatively compared to how others perceive them. This “better than average” illusion has been amply demonstrated (Brown, 2012) in driving, learning, teaching, parenting, health, work competences, leadership ability, remembering better than others and in even having more intelligent and gifted children than neighbors or friends. For instance, 94% of US professors rated themselves better than their colleagues (Cross, 1977), 80% of drivers regard themselves above average on a number of important characteristics (McCormick et al., 1986), only 1% of Australian workers rate their job performance as below average while 11.5% believe they are below average in keeping fit and healthy (Headey and Wearing, 1989). Subjects even rated themselves better than others in predicting the sequence of coin tosses (Langer and Roth, 1975). The better than average illusion has also been called the “Lake Wobegon” effect (Keillor, 1985; Kruger, 1999), named after a fictional location, where “all the women are strong, all the men are good looking, and all the children are above average,” describing a real and pervasive human tendency to overestimate one’s achievements and capabilities in relation to others. On the opposite side people tend to undervalue dangers such as disease, serious accidents and other hardships whose existence they accept but believe cannot happen to themselves (Dolinksi et al., 1987; McKenna, 1993).

On the affirmative side, Taylor and Brown (1988, 1994) claimed that positive illusions are adaptive, enabling people to feel hopeful in the face of great difficulties and overwhelming uncertainty. Positive attitudes can contribute to the achievement of tough tasks, like starting a new firm that should not have been undertaken if objective logic had been applied, to work harder, to be more productive and not give up in the face of hardship. Taylor and Brown (1994) assert that *“most people exhibit positive illusions in three important domains: (a) they view themselves in unrealistically positive terms; (b) they believe they have greater control over environmental events than is actually the case; and (c) they hold views of the future that are rosier than base-rate data can justify”* (p. 21). Moreover in another article Taylor and Brown (1988) claim: *“a set of interrelated positive illusions – namely, unrealistically positive self-evaluations, exaggerated perceptions of control or mastery, and unrealistic optimism – can serve a wide variety of cognitive, affective, and social functions”* (p. 193).

Another article by Taylor and Armor (1996) provides a basis for integrating positive illusions with the constraints of reality and answers the question “Do higher levels of positive illusions predict higher levels of adjustment?” Although, the advantages of positive illusions have been criticized, newer evidence from the extensive literature of positive psychology (DeSalvo et al., 2006; Bushwick, 2012) portrays the advantages of positive thinking that extend from greater happiness (Seligman, 2003; Gilbert, 2005) to longer life expectancy (Bopp et al., 2012). There is also evidence on the benefits of so-called expressive writing, that it can improve mood disorders, help reduce symptoms among cancer patients, improve a person’s health after a heart attack, reduce doctor visits, and even boost memory (Parker-Pope, 2015). Although positive illusions seem beneficial in many cases, the critical question is the extent of their value and whether or not the derived benefits exceed the negative consequences of unrealistic expectations and their possible psychological and financial harm in case of failure. Research, however, has shown (Taylor and Shepperd, 1998) that people occasionally will be pessimistic; bracing themselves for negative feedback, if they anticipate that their optimistic out-look might be challenged. In addition, research by cultural psychologists has revealed (Heine and Hamamura, 2007) that positive illusions are likely to vanish in East Asian populations or they tend to be of different nature (Endo et al., 2000).

The best documented research related to positive illusions comes from the area of self rated health (SRH) where participants are asked a variant of the following question:

How would you describe your state of health in general?

• Excellent
• Quite good
• Fair
• Rather poor
• Very poor
• I don’t know

**FIGURE 1:**
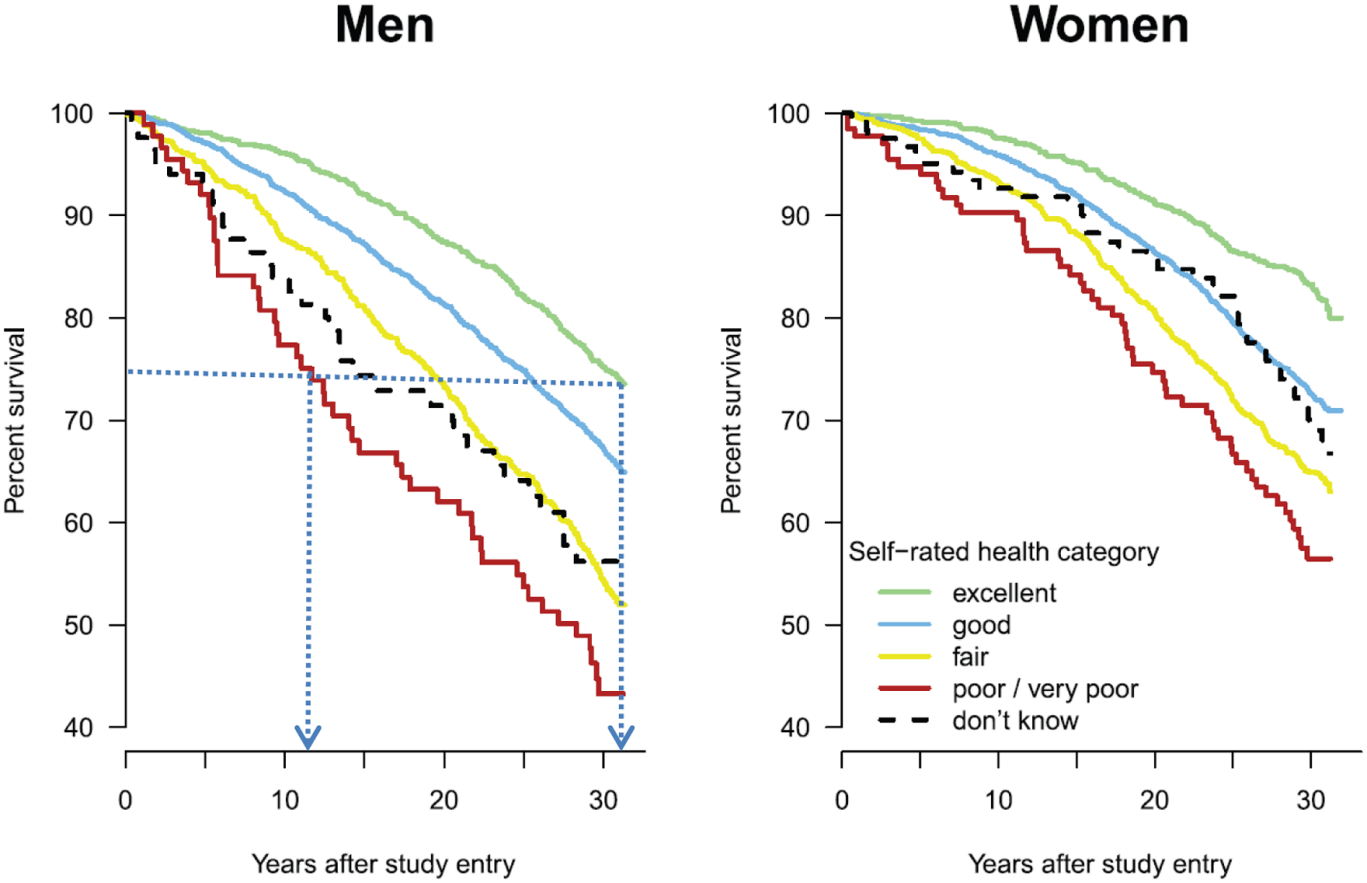
**Survival of men and women by self-rated health category (Bopp et al., 2012), Switzerland 1977–1979, followed up until 2008 (the dotted lines which show a difference in survival of about 20 years between male participants answering “poor/very poor” and those answering “excellent” have been added by the authors)**.

In many studies (Hoorens, 1993; Jylha, 2009; Kawada et al., 2009; Bushwick, 2012) it was found that SRH is a strong predictor of mortality and gives a more accurate estimate of life expectancy than that made by doctors after a medical examination and a study of the medical history of the same people who have answered the SRH. What the SRH studies have shown is that people who feel better (are positive) about their health tend to live by as much as 20 years longer than those who do not [see **Figure 1**, taken from Bopp et al. (2012)]. This type of evidence shows that optimistic thinking and positive illusions need to be considered for their contribution to our well-being and also how their value can be exploited.

According to a recent SRH study administered to more than 8,000 individuals in Switzerland (Bopp et al., 2012) it was concluded that the association between optimism and mortality was *“largely independent from covariates and remained significant after decades… suggesting that SRH provides relevant and sustained health information beyond classical risk factors or medical history”* (p. 1). **Figure 1** shows the significant differences between the various answers to the question of the perceived state of health of the men and women who participated in the survey and speaks by itself on the value of positive disposition.

Results as those of **Figure 1**, as well as those of the other SRH studies, support Taylor’s claims that the illusions of control are promoting rather than undermining good mental health. Although excessive illusions can also be detrimental, it can be argued that they are preferable to their opposite, that of *learned* helplessness (Seligman, 1975; Nolen, 2014) when people give up trying because they believe that future events cannot be influenced by them in any constructive way. Ideally, there should be some optimal level between positive illusions and helplessness depending on each specific situation and the benefits and harm associated with each. According to the opinion of the authors, positive illusions, however, seem beneficial to probably the most important human objective, that of a longer life expectancy while also contributing positively to other aspects of human endeavor.

## The Negative Consequences of Positive Illusions

In addition to their benefits, positive illusions can also lead to harmful consequences. For instance, the effort put in a job can decrease if an employee, believing he/she is better than average, does not put in additional effort to compete with his or her co-workers. The same is true with car accidents that could have been avoided if the driver had paid more attention by not believing he/she has superior skills versus those of others. Similarly, by setting unrealistic goals driven by overconfidence there is the danger that they could not be achieved resulting in unpleasant surprises and unnecessary failures thus leading to financial and psychological harm. In addition, serious monetary losses can occur, for instance, by believing that gains in casinos are possible through good luck while under valuing the odds favoring casinos (illusion of control). People should at least be aware of the negative implications of unrealistic optimism and believing that they can control chance events, in particular when planning and dealing with future oriented situations where predictions for forthcoming, uncertain events are required and the illusion of the planning fallacy prevails (Flyvbjerg et al., 2003; Lovallo and Kahneman, 2003).

Psychologists have documented the *“planning fallacy”* (Buehler et al., 1994; Lovallo and Kahneman, 2003) based on over optimistic predictions that result in cost and time of completion overruns and overestimates of the expected benefits. There are numerous, prominent examples of this planning fallacy. The *Sydney Opera House* (see Sydney Opera House, n.d., where n.d. stands for no date available) was, for example, scheduled to finish in 1963 with a budget of $7 million. Instead it was completed 10 years later, costing 15 times the original estimate. The same situation has been true with most mega projects. The *Eurofighter* (Sanna et al., 2005) took 6 years longer to complete than originally estimated with a cost overrun of €8 billion. Similarly, the final cost of the *Channel Tunnel* (BBC News, 1990) was close to double the original budget with a delay of more than a year in completing it. Even worse, the estimate of the number of passengers to use the *Eurostar* (Eurostar History, n.d.) train service was highly optimistic resulting in a serious shortfall in revenues and heavy losses. In 2014, 20 years after its inauguration the Eurostar has still not managed to carry the passengers predicted in the original forecasts and has made a small profit only in the last 2 years (BBC News, 2015).

In a book Flyvbjerg et al. (2003) shows that large cost overruns have occurred in nine out of 10 large public works projects documenting overruns of 45% for rail projects, 34% for bridges and tunnels, and 20% for roads. Interestingly, the overruns have been constant for the 70 years for which data is available. This may indicate that no improvements in estimating and managing costs have been made over time, or it may be due to other factors. Finally, there are serious concerns that cost underestimation appears to be deliberate in many cases. Flyvbjerg et al. (2003), therefore, criticize professional planning organizations for not doing much to reduce the size of costs overruns and improve the planning process by utilizing a more objective approach that avoids the overconfidence biases and is not based on illusory thinking.

There have been many explanations about the reasons involved in overestimating benefits and underestimating costs and completion times and several suggestions to achieve more realistic estimates have been advanced (Flyvbjerg, 2014). These suggestions range from automatic budget increases to adopting an outside view, or making estimates based on different perspectives (Lovallo and Kahneman, 2003). However, such suggestions have not been implemented in practice according to Flyvbjerg (2011) who as the title of his chapter claims “over budget, over time and over and over again” seem to continue despite all efforts to improve the management of major projects. In the next section the challenge of quantifying the costs of positive illusions, with emphasis to that of control is undertaken in five areas while also providing some suggestions as to what can be done to reduce their negative consequences.

## Quantifying the Costs of Positive Illusions

As it has already been mentioned, there are benefits from positive illusions but there can also be some grave costs. In this section, an effort is made to quantify such costs in order to comprehend their enormous magnitudes and potential damages and also to induce people to avoid them.

### Gambling and Casinos

The gross global winnings (total take minus pay-outs, excluding expenses) from legal global casino gambling comes to the staggering sum of close to half a trillion dollars a year (The Economist, 2014) which is about the GDP of Norway and higher than that of the majority of countries. Moreover, the per capita gambling losses in some countries exceed $1000 a year and can create serious problems to family budgets and individual debts. Can such huge losses be rationally explained? The answer clearly depends on the level of gambling. A few sporadic visits to casinos are not the same as frequent playing or pathological gambling. In the first case it can be done for its entertainment value and the thrill of winning. The problem comes from recurrent playing as it is well-known that the longer one gambles, the chances of winning diminish proportionally to the number of “plays” and the type of gambling as there is a different “house edge” for each type favoring the casino. Such “edge” makes it impossible to win in the long run.

In casinos the cost per play can be calculated precisely and the total losses, depending on the number of plays, can be estimated exactly (see for example the site Get Gambling Facts, n.d.). Such costs range from a loss of 11% when playing “the wheel of fortune,” to 9% each time playing slot machines, to a 2% when playing black jack and a mere 1.4% in “craps tables.” The cost of playing roulette with one zero is 2.7% and that of roulette with two zeroes is 5.4%. These costs mean that every time an individual is betting $1 in a “wheel of fortune” he or she is losing 11 cents on average while the equivalent loss when playing roulette with one zero is 2.7 cents. Things are more complex in Black Jack as the loss of 2% assumes a perfect knowledge of the game and that no mistakes are made, the cost can be much higher otherwise. The least losses occur in “crap tables” at 1.4 cents to each dollar bet. At the same time craps is not a popular game either because the excitement of playing this game is lower or because people are not well-aware of the odds involved. Independently of the size of costs the best strategy is to gamble as few times as possible as it is extremely hard to win in the long run.

Some assert that spending time in super luxury casinos with five star restaurants, super shows and exciting surroundings is their main motivation for gambling on the side. But is this true given the massive level of casino profits that come from players’ losses? For most gamblers the major motivation is the thrill of winning based on the illusion that this is possible and that good luck exists and can be on their side as their belief in the illusion of control motivates them to gamble. Some people believe they can apply a winning strategy that would beat the casino. For instance, they bet the color of the next roulette ball as black after they have observed that, say, five reds in a row have occurred (this is known as the gambler’s fallacy). Similarly, they believe that they can win by betting on their “lucky” number, or that they can correctly guess other purely chance outcomes. Clearly, the cost of such illusions is huge as there is no way to beat chance events, no matter what the strategy being used.

Barring illusions and accepting that no one can win in the long run, what is the best strategy to minimize one’s losses? Obviously, it is not playing “wheel of fortune” or slot machines while the smallest losses occur in “craps tables” which, however, do not seem to be very popular. The losses in black jack are also low but one must be perfectly familiar with the game in order to avoid technical and/or psychological mistakes. The other choice is playing roulette with one zero that does not require any skills, as winning or losing is purely a matter of chance and the “average loss” is small in relation to some other games. The best approach in all casino games would be to bet the entire amount available into a single, or few, plays and then stop whether winning or not. The problem with such strategy is that it eliminates the “excitement” of playing in the expectation of big wins. However, it minimizes the “house edge” as it avoids the cost of many plays. The rule of playing until losing a fix amount is invariably suboptimal as the cost of each additional play favors the casino with the result of an inevitable loss in the great majority of cases.

What is the magnitude of casino gambling costs due to positive illusions and that of control more specifically? According to the Economist article they are huge. Worse, such costs are often paid by people who cannot afford it resulting in negative financial consequences for their families and themselves.

### Stock and Other Markets

There is a big difference between gambling and stock markets. In the former, as we saw, it is impossible to win in the long run while in the latter the exact opposite is true. The long run holds gains for the patient investor who is not influenced by the illusion of control, believing that profitable stocks or other investments can be chosen and/or that the right timing to buy or sell can be predicted. Considerable research has shown that such beliefs are illusory while it is well-known that the stock market, at least in advanced countries, grows consistently over the long run (ranging from 7.4% in Australia to 1.8% in Belgium with a world average of 5.4% during the period of 1900–2001, Dimson et al., 2002). However, it must be understood that long term may mean more than a decade in some instances, making it necessary to be patient and not be influenced by short term fears or greed (Makridakis et al., 2010).

In the short term the cost of market losses can be many times higher than that of casinos. During the 2007–2008 Great Recession, global stock market losses exceeded $30 trillion (World Federation of Exchanges, n.d.) with a sizable part coming from small investors who buy late during periods of booms and sell just before the end of the downturns. Such a number comes to the colossal sum of $17,500 for each person on earth. In addition, there were similarly high losses related to positive illusions, and more specifically to that of control, in other markets than that of stocks. This is particularly true in real estate but also in commodity markets. It is not, therefore, uncommon for some people to lose huge fortunes in the hope of greater returns by downplaying or ignoring uncertainty and risks associated with short term investing, or better gambling in the belief they can predict short term movements in these markets.

Stock and other markets are unpredictable in the short and medium term while there are two types of illusions affecting investors. First, they believe that they can correctly predict, say profitable stocks or the right time to buy or sell, events that are actually random, or very close to that. Alternatively, they assume that someone else, an expert can do that for them. Considerable research has indicated, however, that neither investors nor experts (Sommer, 2015) can outguess the markets which behave in a random “walk” fashion-meaning that the best prediction for the future is today’s price, as postulated by the efficient market hypothesis (Fama, 1970) Believing the opposite is an expensive illusion that can result in great losses that become worse during periods of recessions or financial crises.

Unlike casinos, stock and other markets can produce significant gains during periods of boom and consistent positive returns in the long run. From March 2009 until the middle of 2015, when this paper was written, the stock market reached new records erasing all past losses and more than triple in value during this period (the S&P 500 was 2,130.8 on May 21, 2015 versus 676.5 on March 9, 2009). The question and big challenge is how to retain as much of the gains and minimize the losses during periods of downturns. A strategy exploiting the long term is what is called “buy and hold.” Assuming that the investor is not obliged to trade his or her securities for liquidity reasons, he or she can avoid selling over influenced by fear or other psychological bias that can lead him or her to irrational actions. Such strategy coupled with a well-diversified portfolio (that is assuming that the right stocks cannot be selected and should be chosen randomly) is the optimal way to invest and can be implemented by buying exchange traded funds (ETFs; Bogle, 2015), that are built assuming inability to predict both the best market timing as well as the most appropriate stocks.

### New Firms and Startups

Perhaps starting a new firm demonstrates more than any other area the advantages and costs of illusions. The percentage of firms that enter and exit the market each year in some countries exceeds the 10% of all firms; according to the (SBA Office of Advocacy, 2014), about 10–12% of firms with employees open each year and about 10–12% close. This means that about a 10th of firms fail each year. This is a huge discomfort for the owners and employees of these firms. On the positive side there is renewal and creative destruction (Schumpeter, 1950) as the new replaces the old. The WSJ Blog (2009) provides a lot of information about new firms and the chances of success, and makes reference to the site “Start Up Nation” (Start Up Nation, n.d.) that specializes on startups, providing advice about the various factors requiring consideration in order to increase the chances of success. It lists 30 questions some of which are the following:

• Have you determined how to compete in the market place?
• Have you ensured that you are price competitive?
• Have you verified that the market is big enough?
• Have you tested your marketing and sales plan?
• Is your intellectual property fully protected?
• Have you estimated your breakeven?
• Have you determined your competitive advantages?
• Have you secured sufficient funding?
• Have you built your Website?

By answering these questions the site comes up with a probability that provides a more objective assessment of the chance of success.

Practically, all new firms start small and only a small percentage manage to survive and grow over time, some to even great sizes. The majority of new firms, however, fail to survive more than a few years obliging their owners to stop operations or go into bankruptcy. Given the small chance of success does it make sense to start a new firm? What can be asserted is that without over optimism few would dare to start a new firm if they knew the high chance of failure. But in practically most cases, such chance is downplayed or ignored even by founders who have started and failed before and who know well the chances of failure. Unrealistic optimism is the foundation on which new firms are established. For the owners who fail, positive illusions are detrimental as they have to face the financial and psychological harm of bankruptcy. For society as a whole, however, positive illusions are beneficial as they fuel creative destruction and add to the collective progress, increasing the rate of employment and the offering of new innovative products and services. Just consider three newly founded firms, Google, Facebook, and Alibaba. Their market capitalization approached $815 billion on May 19, 2015 (Google Finance, 2015). This amount probably compensates for all firms that have failed while at the same time these three firms have provided new, innovative services to the whole world. Without over confidence people would have been deprived of Googling, being able to keep in constant touch with friends and acquaintances or trading safely with the world using Alibaba. These benefits compensate for the costs of individual firms failing and the hardship of their founders.

Societal progress requires creative destruction to replace the old with the new. Founders of new firms are extremely useful as they contribute to such creative destruction. To minimize or avoid the harm from failure, founders must be aware of the odds of failure so that they should not get caught by surprise and instead adopt the right mentality not feeling ashamed if they become bankrupt but consider that they have gained valuable experience that can become useful in the future. In addition they should have a plan B so that they are not totally financially ruined. In our days founders are not required to put down their own money or that of family/friends to start their business as venture capital and sites like *Kickstarter* (Kickstarter, n.d.) can help finance promising startups.

### Preventive Medicine

Are the potential benefits of preventive medical tests greater than the harm (including monetary costs) they may cause? In this section we will answer this question by concentrating on three preventive tests: general checkups, mammography for women, and prostate tests for men. We will show that preventive medicine is another illusion which makes people believe they can defeat disease and extend their life expectancy, but in final analysis it causes more harm than benefits overall.

Yearly checkup examinations started in the early 1920s and have continued since then, although many studies going back to the 1960s have shown no benefits from them (Mehrotra et al., 2007). According to the independent Cochrane Foundation (Krogsboll et al., 2012) *“General health checks involve multiple tests in a person who does not feel ill with the purpose of finding disease early, preventing disease from developing, or providing reassurance…. To many people, health checks intuitively make sense, but experience from screening programs for individual diseases have shown that the benefits may be smaller than expected and the harms greater. A possible harm from health checks is the diagnosis and treatment of conditions that were not destined to cause symptoms or death”* (p. 2). The final conclusion of the authors of this study is:

*“General health checks did not reduce morbidity or mortality, neither overall nor for cardiovascular or cancer causes, although the number of new diagnoses was increased”* (p. 2).

Yet, despite evidence against routine annual examinations, many family physicians recommend them (Mehrotra et al., 2007) exploiting the “illusion of reassurance” that a preventive test will catch health problems early, reduce disease and increase life expectancy. According to these authors the cost of the “illusion of reassurance” in the USA has been estimated at around $8 billion a year as about 8% of the doctor visits in the USA are for annual checkups. In addition to the monetary cost, there could be additional harm caused by checkups that include pointless radiation and over treatment resulting in unnecessary medical actions (Consumer Report, 2012). In his NYT article (Welch et al., 2007) doctor Gilbert Welch and his coauthors explain how test results make people sick and why visiting a doctor can be hazardous to your health. Their advice is to avoid all preventive medical care.

According to O’Donoghue et al. (2014) annual mammography tests were recommended for all women older than 40, with 70% of all USA women participating at an annual cost of $7.8 billion in 2010. O’Donoghue et al. (2014) assuming different rates of screening, starting age and 1 versus 2 years intervals, estimated costs that ranged from $2.6 to $10 billion annually. Today, however, there is an intense debate regarding the value of any screening, at any cost, with those opposing it saying that the potential harm is greater than the benefits (Science Newsline Medicine (2015). Gøtzsche in his book (Gotzsche, 2012) “Mammography Screening: Truth, Lies, and Controversy” states:

*“If we wish to reduce the incidence of breast cancer, there is nothing as effective as avoiding getting mammograms. It reduces the risk of getting breast cancer by one-third.”* and later he continues “*We have had devastating epidemics of hysterectomies and tonsillectomies and we currently have an epidemic of Cesarean sections, although these operations may lead to substantial harm. We have also epidemics of mastectomies and prostatectomies because people seem to be more worried about dying than they are interested in living*.” (p. 349)

Mammography screening also requires a huge monetary cost that could be spent on more beneficial causes (e.g., cancer research). The actual dollar cost of screening in the USA was $7.8 billion in 2010 (O’Donoghue et al., 2014) and is estimated that such cost will exceed $10 billion if all women between 40 and 84 years-old are tested. However, it can be reduced to $2.6 billion if it is restricted to women of 50–69 and it is done biannually (O’Donoghue et al., 2014). Gotzsche (2012) in his book cites a paper by Wainer (2011) which goes beyond monetary estimates and calculates the overall cost of extending one life by mammography tests at $329,000 if the harm of screening is included.

Prostate cancer tests for men are also common as it is estimated that close to 52% of men are tested annually at a cost reaching half a billion for Medicare patients alone in the USA (Wang et al., 2014). Richard Ablin, the inventor of the PSA test used to diagnose prostate cancer, in his book (Ablin, 2014) The Great Prostate Hoax states:

*“The ability of the PSA test to identify men with prostate cancer is slightly better than that of flipping a coin. And its continued use as a routine screening tool is nothing short of a national health disaster”* (p. 6). And later he continues *“Among the 1,000 men who had the PSA test, 20 of them would have radical prostatectomies for cancers that never would have caused symptoms. And five of those men would have lifelong complications, including impotence and incontinence.”* (p. 45)

Later in his book, Ablin writes that if the probability of men dying from prostate cancer is 3% a year, that of surviving a diagnosis of prostate cancer is 97% whether he receives treatment or not. Then why should a man be screened if the harm of false diagnosis is 30 to 100 times the estimated benefit, resulting in less than 0.1% reduction in prostate mortality over 10 years? His conclusion is that the screening is done because of financial interests as it increases the number of additional tests and prostatectomies. He asserts:

*“Without radical prostatectomies, more than half of all the urology practice in the US would go belly-up.”* (p. 42)

A lot of research has shown that the potential benefits of preventive medicine are illusory and that the harm involved is considerably greater than whatever the advantages. Unfortunately those who profit from medical checkups and screening exploit the human psychological need to reduce future uncertainty by the false claim that preventive medicine identifies potential health problems early so that they can be dealt with before they become more serious. The monetary cost and the additional harm, however, is much greater than the benefits requiring a rational approach and a realization that it is illusory to believe that future disease can be predicted correctly and consequently that actions can be taken to improve one’s chances of a healthier and longer life expectancy. Objective research has shown that uncertainty cannot be eliminated by preventive medicine that has an opposite effect than that intended, and causes much more harm than gains. Nature’s predispositions about our health cannot be predicted. The uncertainty of when disease is likely to strike and when death will occur cannot be eliminated with any degree of certainty (the exception being our own feeling of how we feel, see section on SRH above) at least at present with preventive medical tests. The critical question of what can be done is considered in the concluding section.

### Wars

Wars are, indeed, influenced the most by positive illusions at a massive cost to the taxpayers and with grave human suffering. In this section, the cost of three wars (Vietnam, Afghanistan, and Iraq) will be appraised and the optimistic predictions of victory leading to these wars will be presented. In his 2015 State of the Union Address, US President Barack Obama asked Congress to authorize military action against Islamic State extremists, saying the US can defeat them without being “dragged into another ground war in the Middle East” (NBC News, 2015). Ironically the Islamic State of Iraq (ISI) was created after the “end” of the Iraq war that was started by President Bush in March 2003 (Sanger and Burns, 2003). How reliable is Obama’s assertion that the Islamic State will be defeated? An aide to former Defense Secretary Donald Rumsfeld confessed that “if we had had the foresight to see how long it would last and even if it would have cost half the lives, we would not have done it” (Trotta, 2013). The Iraq war was declared based on (a) optimistic assessments of an easy victory, (b) that Iraqis would consider the US and allied troops as “liberators” and (c) on the false belief that Iraqi dictator Saddam Hussein’s government held weapons of mass destruction (after exhaustive search no such stockpiles were found). The reality proved quite different as described by Stewart (2007), who talks of the anarchy that begun just 6 months after the start of the war.

A NYT article estimates that the US war in Iraq has cost $1.7 trillion with an additional $490 billion in benefits owed to war veterans, a figure that would grow to more than $6 trillion over the next four decades (Trotta, 2013). This article also asserts that the war has killed at least 134,000 Iraqi civilians and may have contributed to the deaths of as many as four times that number without counting security forces, insurgents, journalists and humanitarian workers. In addition, the US medical and disability claims for veterans after a decade of war totaled $33 billion while 2 years later, that number had risen to $134.7 billion. Worse still, it estimates that such claims will exceed the billion dollar mark at the end of the second decade. These estimates indicate a huge monetary and humanitarian cost with practically no benefits as the ISI would not have existed if Saddam Hussein had stayed in power. Moreover, the objective that the war would have allowed the USA to control Iraq’s oil never happened. As US firms left the area because of increased insecurity, Iraq started selling a sizable and growing percentage of its oil production to China which is providing drilling technology and capital investments to the country. If current trends continue, China will end up “owning” a good part of Iraq’s oil production facilities without having to fight a multitrillion dollar war (Arango and Krauss, 2013).

The Iraq war was not the first to cost trillions of dollars. The war in Afghanistan started in October 2001 with the goal to drive the Taliban from power, because they had allowed Osama bin Laden and al Qaeda, to hit the World Trade Center in the 9/11 catastrophe. Ironically, to fight the fundamentalists that the USA and Saudi Arabia had trained and financed to combat the Soviets who had invaded Afghanistan in December 1979. It is estimated that the US and Saudi Arabia provided more than $40 billion worth of weapons and money to the fundamentalist Mujahideen over the course of their war against the Soviets, with part of the money used to set up thousands of fundamentalist Islamic religious schools (Madrassas) for the Afghan refugee children flooding into Pakistan that consequently became the major recruiting basis for the Taliban (Visalli, 2013).

The Americans believed that they could do better than the Soviets, and the British before them, in achieving their objectives in Afghanistan which according to Obama (DeYoung, 2009) included (a) “create a country stable and strong enough to prevent al-Qaeda from reoccupying Afghan” and (b) being able “to disrupt, dismantle and defeat al-Qaida.” Reality shattered their “irresistible illusion” as it is shown in an article in the London Book Review (Stewart, 2009). The USA was obliged, like the British and the Soviets before them, to leave Afghanistan 13 years later, at the end of 2014, after having paid a huge price both monetarily and in human suffering. The longest overseas history war has cost the USA alone, one trillion dollars and it would require spending several hundred billion dollars extra in the future for the 10,000 soldiers remaining in the country and medical and disability expenses for war veterans. These costs do not include interest payments for the increased debt caused by the Afghan war or the extra pension expenses of the military associated with this war. The human cost of the war has been more than 20,000 dead soldiers and contractors, almost 100,000 injured and with medical problems and 875,000 disability claims. Sadly, no one, even the strongest supporters of the war, has claimed any benefits.

The war in Vietnam is another sad story with an extraordinary cost, the illusion of great benefits and no actual ones. At the time the loss of South Vietnam to the communist North was feared by US policy makers that it would create a domino effect for other countries like Laos and Cambodia but then also Thailand, Malaysia, Singapore, and even Indonesia to fall next under communist domination. Most ironically, it is communism that has totally collapsed and countries like China and Russia that were supporting Vietnam in its war with the USA have abandoned communism and seen the greatest increase in their number of billionaires. Furthermore, Vietnam and China have become bitter enemies as both are claiming part of the South China Sea thought to contain rich oil reserves (LSE Ideas, n.d.).

US involvement in Vietnam started in the 1950s, not long after the French left the region. It was increased in the early 1960s and was elevated when regular US combat units were deployed in 1965 (Alpha History, n.d.). It was expanded into Laos and Cambodia and was peaked in 1968 when North Vietnam launched the Tet Offensive that failed in its goal of overthrowing the South Vietnamese government but convinced a large segment of the US population that winning the war was illusory despite the massive US spending and the heavy losses in dead and injured.

The monetary cost of the war has been estimated to around $145 billion (more than one trillion constant 2015 dollars) without including medical and disability benefits and interest costs that probably double the actual outlays. There is also huge human suffering. It is estimated that almost 60,000 US troops were killed, more than 150,000 were wounded, 23,000 suffered total disability and 2,000 were missing. A worse statistic was the number of 70,000–300,000 Vietnam Veterans who committed suicide and another 700,000 veterans who suffered psychological trauma because of the atrocities of war they experienced. On the South Vietnam side the number of dead was 400,000 and on the North 900,000 (How Much Did the Vietnam War Cost?, 2014).

The illusion of underestimating the real factors surrounding a war and the optimistic assessment that the situation can be controlled involves an immense cost and no benefits, at least in the three wars covered in this section. Yet, intelligent and well-meaning people believe otherwise causing grave suffering and wasting a large amount of money that could have been used to improve the lives of a great number of people in their country.

## Conclusion and Directions for Future Research

This paper has considered gambling, investing/speculating in stock and other markets, new firms and startups, preventive medicine as well as wars as prime examples influenced from positive illusions. It has shown that accepting uncertainty and avoiding illusions encompasses substantial benefits and ensues in huge reductions in monetary costs and harm in general, although there could be some undeniable benefits from some positive illusions. What we must do is exploit the benefits of positive illusions while avoiding their costly, negative consequences even though psychologically this is difficult. Ideally, we should be able to strike a balance between the benefits and costs by estimating them for each case as accurately as possible.

We live in an uncertain world and we must accept living with the ensuing uncertainty. It is clear that uncertainty creates anxiety and involves risks. Some uncertainties are easy to deal with. Whether or not it will rain tomorrow can be dealt with by being dressed appropriately and having an umbrella, thus eliminating the risk of getting wet. If it does not rain, the cost of being prepared is small and acceptable (weather forecasters overestimate the chance of rain on purpose for this reason). The uncertainty of investing in the stock market and the risk of losing money is more serious but not investing could include a significant opportunity cost if the market goes up. It is possible, however, to balance the two risks (investing and the market goes down and not investing and it goes up) if the right type of investment strategies are followed although investments can never be risk free. The possibility of becoming seriously ill is another uncertainty with much more serious consequences as the risk in such case is huge and may even lead to death. Ironically the best strategy in this situation is to avoid preventive medical screening and not even go close to a doctor unless feeling sick. Even though such strategy seems counter intuitive, it must be followed as the harm of preventive screening is much greater than all their possible benefits. The challenge is, therefore, to be able to figure out the benefits and costs objectively and quantify them where possible, instead of illusory believing that preventive medicine can reduce our chance of disease and increase that of our life expectancy.

Gigerenzer, in his book (Gigerenzer, 2014) advocates that we must become “risk savvy” by getting rid of the illusion of certainty, that is the belief that something is sure when actually it is not. He writes:

*““Savvy” means, acute, astute, and wise. But being risk savvy is more than being well-informed. It requires courage to face an uncertain future as well as to stand up to authority and ask critical questions.”* (p. 15).

Avoiding the illusion of certainty and not falling victim to the huge cost associated with positive illusions would require the following:

(1) Avoiding the bias to believe that chance events can be controlled and attribute our success to our own skills while blaming bad luck for our failures. This can be best achieved by attempting to figure out uncertainties correctly in the form of probabilities and accurately assessing their long term impact.
(2) Understanding that when playing in casinos and using preventive medicine there is no way that the benefits will be greater than the costs on average and in the long run. When investing in the stock markets of advanced countries, on the other hand, the long term is associated with profits, at least until now.
(3) When considering the gains and losses of new firms or startups we need to investigate them on two levels: that of individuals and that at the aggregate, social level. At the individual level, the great majority of new firms/startups fail. At the aggregate level, however, there are great benefits, even if few firms survive and thrive. For instance Google, Facebook and Alibaba’s, market capitalization can probably compensate for the cost of all new firms/startups that failed. This may not be a comfort for the individuals who have been ruined financially but it adds to societal progress and benefits humanity as a whole.
(4) Realizing that for personal or professional interests some experts do their best to persuade us that they can reduce future uncertainty and that we can benefit from their advice and/or actions. This is not true as many studies have shown. Experts cannot outperform well-informed individuals or predict better than them. This is also true in the domain of medicine where doctors cannot improve your chances of a healthier or longer life by preventive measures. The SRH studies have come to two extremely important conclusions. First, that doctors cannot predict more accurately than us how long we will live and secondly, the most important factor for a long life is our own disposition today.
(5) Exploiting the benefits of positive illusions to improve our positive thinking and our psychological mood is critical in many situations and can lead us to be better motivated and persevere when dealing with hardship.
(6) Avoiding the negative consequences of positive illusions or minimizing their magnitude can be done by distinguishing between the short and long term effects of various decisions/actions and in particular realizing the implications of the illusion of control by accepting that it is impossible to control chance events and gain from them in the long run.
(7) Some additional actions are shown in a footnote at the end of this paper^1^.

Future research must follow four directions. First, more work is needed to be able to quantify the cost of various illusions in more areas than the five covered in this paper. This can be done by providing incentives, in the form of research grants, by government agencies as well as non-profit organizations and even medical insurance firms. Doing so could substantially reduce the costs of medical care by making evident the cost of illusions. Second, much more is needed to find more effective ways to communicate the huge cost of illusions and educate people to become “risk savvy” by better understanding the benefits and harm of illusions. It is not enough to write academic articles and books. Great effort on effective advertising will be needed to do so, even though there will be extensive opposition from well-organized and thoroughly funded lobbies. Third, it would be important to devise and communicate practical strategies of how people can avoid falling victims to positive illusions while also exploring their benefits. This is particularly true in medicine where the stakes are as vast as the profits of big pharmaceutical companies and doctors, exploiting people’s illusions, are in the hundreds of billions each year. Finally, the number of “brave” people writing books and articles exposing the harm done by preventive medical screening must be multiplied and written not for scientists but for the general public who mustunderstand the extent of the harm involved. At the same time the government agencies regulating the pharmaceutical companies and the doctors’ lobbies must become more active, informing the people accurately about the harm of preventive medical screening and saving both hundreds of billions of dollars, as well as millions of lives each year.

## Conflict of Interest Statement

The authors declare that the research was conducted in the absence of any commercial or financial relationships that could be construed as a potential conflict of interest.

## Footnotes

1 It is critical to utilizing independent sources to obtain information to base our decisions. Some of these independent sources for each of the five areas being considered in this paper are the following:

(a) Gambling and casinos: there are two types of gambling those that depend on pure chance and those that involve a combination of chance and skills. Undoubtedly, it is easier to know the cost of losing in the first case than in the second. For casino playing for instance the site http://getgamblingfacts.ca/cost-of-play/ provides specific information for all casino games and the cost associated with each “play” while Wikipedia provides the probabilities for different types of gambling games including various types of poker, blackjack, dice and others. However, in some of these games psychology, personality and memory may be as important as knowing the probabilities.
(b) Stock and other markets: http://www.swapmeetdave.com/Bible/InvLinks.htm
(c) New firms and startups: http://blogs.wsj.com/venturecapital/2009/06/09/calculating-a-start-ups-odds-of-success/ and http://www.startupnation.com/ and the free lunch assessment it provides.
(d) Preventive medicine: the pursuit of self-serving interests by pharmaceutical firms and doctors providing the advice is not objective. The following is a list of websites from where independent, objective information can be found (most have been taken from Gigerenzer, 2014):
  (i) The Cochrane Collaboration: http://www.cochrane.org/
  (ii) Therapy reviews: http://www.thennt.com/home-nnt/
  (iii) US Preventive Services Task Force, www.ahrq.gov/CLINIC/uspstfix.htm
  (iv) Agency for Healthcare Research and Quality, www.ahrq.gov
  (v) Dartmouth Atlas of Health Care, which shows the wild variability in how doctors treat patients, www.dartmouthatlas.org
  (vi) Bandolier Oxford, www.medicine.ox.ac.uk/bandolier/
  (vii) Foundation for Informed Medical Decision Making, www.informedmedicaldecisions.org
  (viii) Harding Center for Risk Literacy, Max Planck Institute for Human Development: www.harding-center.com
  (ix) A useful watchdog group that rates what’s good and bad in health journalism: www.healthnewsreview.org
(e) Wars: Afghanistan and Iraq http://costsofwar.org/ and Vietnam http://thevietnamwar.info/
